# Chronic hypoxia favours adoption to a castration-resistant cell state in prostate cancer

**DOI:** 10.1038/s41388-023-02680-z

**Published:** 2023-04-05

**Authors:** Sarina Cameron, Genevieve Deblois, James R. Hawley, Aditi Qamra, Stanley Zhou, Seyed Ali Madani Tonekaboni, Alexander Murison, Romy Van Vliet, Juan Liu, Jason W. Locasale, Mathieu Lupien

**Affiliations:** 1 Princess Margaret Cancer Research Centre, Toronto, ON Canada; 2 Department of Medical Biophysics, University of Toronto, Toronto, ON Canada; 3 Duke University School of Medicine, Durham, NC USA; 4 Ontario Institute for Cancer Research, Toronto, ON Canada; 5 Present Address: Institute for Research in Immunology and Cancer (IRIC), Faculty of Pharmacy, University of Montréal, Montréal, QC H3T 1J4 Canada

**Keywords:** Prostate cancer, Transcription, Cancer metabolism

## Abstract

Predicting and treating recurrence in intermediate-risk prostate cancer patients remains a challenge despite having identified genomic instability [1] and hypoxia [2, 3] as risk factors. This underlies challenges in assigning the functional impact of these risk factors to mechanisms promoting prostate cancer progression. Here we show chronic hypoxia (CH), as observed in prostate tumours [4], leads to the adoption of an androgen-independent state in prostate cancer cells. Specifically, CH results in prostate cancer cells adopting transcriptional and metabolic alterations typical of castration-resistant prostate cancer cells. These changes include the increased expression of transmembrane transporters for the methionine cycle and related pathways leading to increased abundance of metabolites and expression of enzymes related to glycolysis. Targeting of the Glucose Transporter 1 (GLUT1) identified a dependency on glycolysis in androgen-independent cells. Overall, we identified a therapeutically targetable weakness in chronic hypoxia and androgen-independent prostate cancer. These findings may offer additional strategies for treatment development against hypoxic prostate cancer.

## Introduction

Prostate cancer is the second most common cancer diagnosed in men, affecting 1 in 5 men on average with a 5-year survival of 98% [5, 6]. Although the 5-year survival rate is high, progression still occurs in approximately 20–34% of prostate cancer patients [7, 8]. Biochemical recurrence (BCR) and the subsequent progression to castration-resistant prostate cancer (CRPC) are the main drivers of prostate cancer-specific mortality [9, 10]. Gleason score, tumour grade, and prostate specific antigen (PSA) levels in the sera are some of the primary means of progression risk stratification for prostate cancer patients [11, 12]. Using these grading systems, patients fall into three main categories: low, intermediate, and high risk [12, 13]. The risk of prostate cancer-specific mortality in high-risk patients is approximately 40% and 4% in low-risk when patient follow-up is extended to 10 years or more [14]. The intermediate-risk patients present the greatest difficulty in predicting cancer progression [6, 15], with a risk of prostate cancer-specific mortality ranging between 6.5% and 11% [14]. Intermediate-risk prostate cancer patients can be subdivided into low-intermediate (Gleason score 3 + 4) and high-intermediate (Gleason score 4 + 3) [5, 6]. While this separation serves to guide treatment selection within intermediate-risk patients, disease progression in the form of biochemical and locoregional recurrence can still occur [15]. These findings reflect the intra-tumour heterogeneity of prostate cancer and the difficulty in predicting disease progression. Therefore, there is a need to identify those patients more likely to develop recurrence and intervene earlier to prevent biochemical recurrence and disease progression.

Beyond Gleason scores and PSA levels, hypoxia [2, 16, 17], genomic instability [1] and the MRI imaging feature anisotropy are recent predictors of prostate cancer progression [18]. While hypoxia is associated with *PTEN* loss [19] and genomic instability in prostate cancer [1, 17, 20], it is also an environmental stressor that can induce non-genetic changes. Hypoxia prevents the degradation of the Hypoxia Inducible Factor 1ɑ (HIF1ɑ) protein, which activates a HIF1ɑ-dependent transcriptional response [21, 22], driving angiogenesis, proliferation, apoptosis and a number of metabolic pathways [23, 24]. These pathways promote survival in a hypoxic environment and are associated with cancer progression [23, 25]. For example, glucose-6-phosphate isomerase (*GPI*) expression is increased in prostate cancer cells exposed to acute cycles of hypoxia (1% oxygen) in an androgen-depleted environment [26]. *GPI* encodes an enzyme important in glycolysis allowing cells to decrease their dependence on oxygen for energy [26, 27]. Hypoxia can also alter the epigenetic landscape of prostate cancer cells by promoting the expression of histone demethylases [28, 29]. For example, the expression of lysine demethylase 3 A (*KDM3A*), a coactivator of AR, is increased under hypoxia, resulting in the decreased methylation of H3K9 and increased expressed of *CTH* and *PFKP* [30–32]. These genes are involved in metabolic pathways, specifically *CTH* in cysteine and methionine metabolism and *PFKP* in glycolysis [30, 33]. Subsequently, hypoxia can alter the epigenetic landscape and give rise to chromatin variants [34] in prostate cancer cells by favouring the expression of KDM3A [28, 29, 31, 32]. Thus, hypoxia represents a potent environmental pressure to promote progression through chromatin variants that impact gene expression and altered metabolic pathways.

Therapeutic interventions designed against hypoxia have become increasingly attractive in solid tumours including prostate tumours [35, 36]. Many hypoxia-based therapies focus on HIF1ɑ [35, 36]. However, HIF1ɑ is primarily involved in acute hypoxia responses, up to 24 h, with a different HIF isoform mediating a longer-term response [24, 37]. The HIF isoforms have different target genes, leading to changes in the transcriptional program [37]. This indicates longer exposure to hypoxia causes further changes in gene expression. Therefore, targeting HIF1ɑ may not necessarily reverse changes mediated by hypoxia. Here we show how chronic, as opposed to acute hypoxia, leads to androgen insensitivity in prostate cancer cells by mimicking transcriptional and metabolic changes seen in CRPC cells. These changes reflect vulnerabilities for targeted intervention using chemical inhibitors of glycolysis to prevent the development of aggressive prostate cancer.

## Results

### Chronic hypoxia promotes insensitivity to androgen depletion

**Fig. 1:**
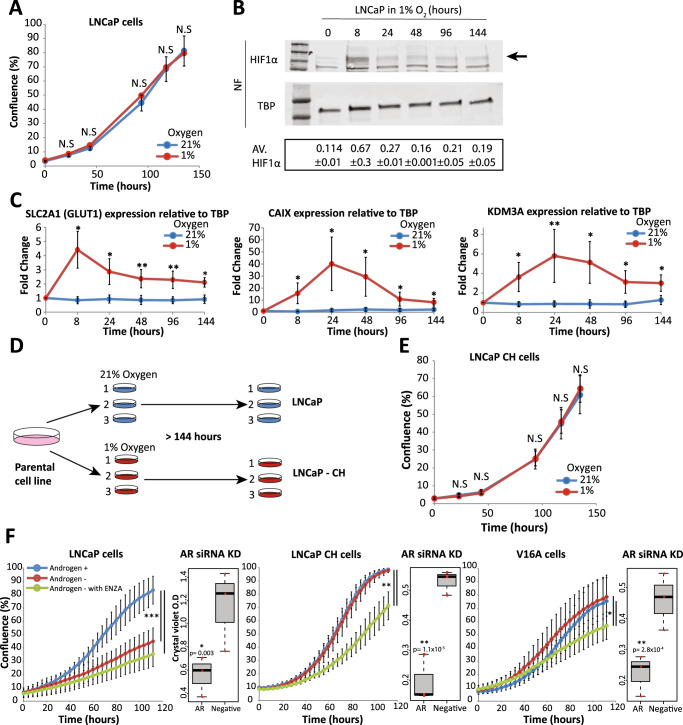
Long term hypoxia exposure promotes androgen insensitivity. **A** Proliferation of the LNCaP cells was measured in either 21% (blue) or 1% oxygen (red), with N.S representing not significant. **B** The hypoxia response in LNCaP cells was assessed by monitoring the protein levels of HIF1ɑ as indicated with the black arrow, normalised to TATA box binding protein (TBP), in the nuclear fraction (NF) of cells exposed to hypoxia with the average signal intensity of biological replicates quantified underneath each timepoint. **C** To confirm the functionality of HIF1ɑ the transcription of the target genes *SLC2A1, CAIX* and *KDM3A* were monitored over time. The samples were normalised to TBP, a housekeeping gene. Unpaired *t*-tests were performed at each timepoint (*p = 0.05–10^−3^, **p = 10^−3^–10^−6^). **D** A schematic of how the chronic hypoxia model was developed, showing the matched biological replicates in either 21% (blue) or 1% (red) oxygen. The chronically hypoxic cells were continuously maintained in 1% oxygen, as indicated by the >144 h. **E** To demonstrate chronic hypoxia didn’t prevent growth, the proliferative ability of the LNCaP-CH cells when reoxygenated (21%) or maintained at 1% oxygen was monitored over time. **F** The LNCaP, LNCaP-CH, and a model of androgen insensitive derived from the LNCaP cells (LNCaP-V16A) were exposed to full RPMI media (Androgen+), RPMI – steroids (Androgen -) and RPMI – steroids + Enzalutamide (Androgen - with ENZA). Unpaired *t*-tests were performed on different media conditions (***p = >10^−6^). The right panel of each graph shows a box plot of the androgen receptor (AR) knocked down (KD) using siRNA with viability measured using crystal violet staining. The inclusion of a negative scramble control (Negative) shows the effect on viability with the knock down.

To study the effect of chronic hypoxia in prostate cancer, the LNCaP cell line, an androgen-sensitive prostate cancer cell line, was continuously cultured in 1% oxygen. To determine whether hypoxia was detrimental to proliferation cells were monitored in 1% oxygen. With no prior exposure to hypoxia, the growth of LNCaP cells was measured for up to 144 h in 21% (ambient) or 1% (hypoxia) oxygen. LNCaP cells showed no significant difference in growth when maintained in 21% or 1% oxygen (Fig. 1A). The ability to grow in 1% oxygen was also demonstrated with VCaP cells, another androgen-sensitive prostate cancer cell line (Supplementary Fig. 1A). In contrast, hypoxia was detrimental to the growth of normal prostate epithelial RWPE-1 and PWR-1E cell lines within 70 h of incubation (Supplementary Fig. 1B). In parallel, we measured HIF1ɑ protein levels in the nuclear fraction (NF) of LNCaP cells to assess the response to hypoxia. In agreement with previous reports [24], HIF1ɑ protein levels increased in LNCaP cells within the first 8 h of incubation in 1% oxygen levels (Fig. 1B). HIF1ɑ levels then decreased but remained present in cells maintained in hypoxic conditions for up to 144 h (Fig. 1B). Expression of HIF1ɑ target genes, such as Solute Carrier Family 2 Member 1 (*SLC2A1*) [23], Carbonic Anhydrase 9 (*CAIX*) [38] and Histone demethylase 3 A (*KDM3A*) [31], showed a marked increase within the first 8–24 h under hypoxia (Fig. 1C). *SLC2A1* and *KDM3A* expression was also demonstrated to increase between 8 and 24 h in VCaP cells (Supplementary Fig. 1C). Although expression decreased over time under hypoxia, mRNA levels for these HIF1a target genes remained above normoxia baseline. Collectively, these results suggest that prostate cancer cells respond to and proliferate unimpeded in hypoxic conditions.

To study the impact of chronic hypoxia on the biological features of prostate cancer cells, we established LNCaP cells from long-term maintenance (>144 h) in 1% oxygen levels (Fig. 1D). We labelled these models of chronic hypoxia (CH). No significant differences were noted in the growth properties of LNCaP-CH (Fig. 1E) when maintained in 1% oxygen versus when reoxygenated and maintained in 21% oxygen. In accordance with the LNCaP-CH cells, the chronically hypoxic VCaP (VCaP-CH) and the LNCaP-V16A cells showed no difference in growth when reoxygenated (Supplementary Fig. 1D, F). As hypoxia is associated with progression in prostate cancer [2, 17], we next assessed the response of our chronic hypoxia models to standard-of-care therapies. Specifically, we tested the growth response of LNCaP-CH to androgen depletion and enzalutamide treatment. Androgen depletion mimics surgical and chemical castration [26]. Enzalutamide is an androgen receptor antagonist commonly used to treat prostate tumours that progress following castration [39, 40]. For comparison, we included the LNCaP and castration-resistant LNCaP-V16A cell lines [41, 42]. Androgen depletion alone (Androgen -) or in combination with enzalutamide treatment (Androgen - with ENZA) abrogated the growth of LNCaP cells compared to control conditions (Androgen+, Fig. 1F). In contrast, LNCaP-CH cells were insensitive to androgen depletion (Fig. 1F). Their growth was reduced only upon combining androgen depletion with enzalutamide treatment. The response of LNCaP-V16A castration-resistant cells to androgen depletion and enzalutamide treatment mimicked the response of LNCaP-CH cells. These findings were also observed in the VCaP and VCAP-CH lines, where androgen depletion had a milder effect on the VCaP-CH line than the parental VCAP line (Supplementary Fig. 1E). However, all LNCaP derived lines remain dependent on the presence of the androgen receptor (Fig. 1F, Supplementary Fig. 1G, H). Suggesting it is not a lack of androgen receptor that allows cells to become insensitive to androgen depletion but another mechanism. Collectively, these results argue that chronic hypoxia favours a castration-resistant-like cell state in prostate cancer cells by conferring androgen insensitivity.

### Chronic hypoxia and castration resistance converge on metabolic pathway disruption

**Fig. 2:**
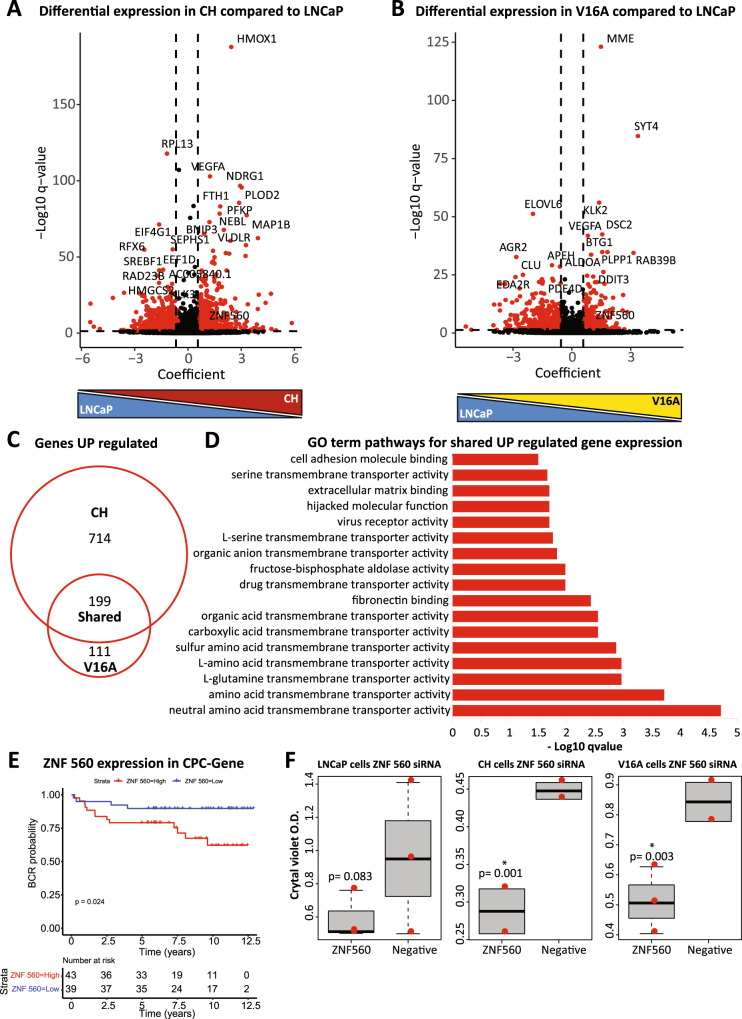
Shared transcriptional changes in the CH and V16As identify metabolic alterations. **A** To investigate expression changes in the LNCaP-CH RNA-seq was performed and the LNCaP-CH cells were compared to the LNCaP cells. The cut-off for significance was a *q* value of 0.05 with the top 50% of the transcripts, as measured by the coefficient, were considered differentially expressed. **B** RNA-seq was also performed in the LNCaP-V16A cells with the same cut-offs that were used for the LNCaP-CH cells. **C** A Venn diagram shows the shared up-regulated transcripts observed in the LNCaP-CH and LNCaP-V16A cells when compared to the LNCaPs. **D** GO term analysis of the shared transcripts (199 genes) identifies metabolic pathway transporters related to glycolysis and the methionine pathway. **E** A Kaplan–Meyer of ZNF560, a transcription factor identified in the shared 199 genes, in the CPC-Gene cohort. **F** Viability was measured using crystal violet staining of the siRNA knock down (KD) of ZNF560 in the LNCaP, LNCaP-CH and LNCaP-V16A cells (*p = 0.05–10^−3^). The inclusion of a negative scramble control (Negative) shows the effect on viability with the knock down.

To better understand how chronic hypoxia could promote castration resistance, we first investigated and compared the expression profile of all three models i.e., LNCaP (androgen sensitive) LNCaP-CH (chronic hypoxia model) and LNCaP-V16A cells (castration resistant) by RNA-seq and identified differentially expressed gene transcripts, based on a *q* value less than 0.05 and the 50th percentile, in both LNCaP-CH and LNCaP-V16A compared to LNCaP. In comparison to LNCaP cells, we identified 913 gene transcripts upregulated and 1252 gene transcripts down-regulated in LNCaP-CH cells (Fig. 2A). We next compared LNCaP-V16A to LNCaP cells, identifying 310 gene transcripts upregulated and 671 gene transcripts downregulated in castration-resistant prostate cancer cells (Fig. 2B). To assess similarities and differences amongst gene transcripts and related pathways in castration-resistant and chronic hypoxic cells we compared the differential expression from LNCaP-CH to that of the LNCaP-V16A cells. We found a total of 532 differentially expressed gene transcripts shared between LNCaP-CH and LNCaP-V16A when compared to LNCaP cells (Supplementary Fig. 2A). In total, 199 transcripts were upregulated and 310 downregulated in both LNCaP-CH and LNCaP-V16A compared to LNCaP cells, leaving 23 genes with divergent expression (Fig. 2C and Supplementary Fig. 2A, C). While pathway analysis from the 310 downregulated transcripts identified only protein heterodimerization in the GO term analysis (Supplementary Fig. 2C). The pathway analysis for both the up and downregulated transcripts did not identify the classical AR pathway even though the viability of the CH and V16A cells remain dependent on the presence of AR. However, pathway analysis from the 199 upregulated gene transcripts, common to both chronic hypoxia and castration-resistant prostate cancer cells, identified metabolic pathways enriched in glycolysis related and metabolite transporter activity including fructose-bisphosphate activity and amino acid transports, glutamine, and serine transporters (Fig. 2D). Further investigation of these pathways using metabolism-specific KEGG analysis identifies hypoxia, glycolysis, and the one-carbon metabolism (Supplementary Fig. 2B). To determine the relevance of the transcriptional profiling in patient samples transcriptional data from the Porto Cohort, patients that did and did not develop BCR, was investigated [43]. The shared 199 transcriptional data set was investigated in the Porto Cohort of prostate cancer patients, where patients are either categorised as “Cases”, those that developed BCR, or “Control”, those that did not develop BCR [43]. Of the 199 transcripts investigated only 166 were covered in the Porto Cohort and separating the samples into Cases versus Controls showed a higher average gene score for those 166 in the Cases with a *q* value of 0.072 (Supplementary Fig. 2F). To identify a regulatory mechanism, we further interrogate the 199 up regulated gene transcripts for the expression of transcription factors (TFs). The top three differentially expressed TFs included SNAI2, ZNF728 and ZNF560 (Fig. 2A, B, Supplementary Fig. 2G). Out of these TFs, ZNF560 was identified as a significant predictor of biochemical recurrence, *p* = 0.024 (Fig. 2E) and had a significant hazard ratio, HR = 3.9 (Supplementary Fig. 2H). Furthermore, knock down of ZNF560 using siRNA in the LNCaP, LNCaP-CH and LNcaP-V16A cell lines showed a significant decrease in viability in the LNCaP-CH and LNCaP-V16 but no the LNCaP cell line (Fig. 2F, Supplementary Fig. 2I). These findings implicate the transcriptional changes identified in chronic hypoxia are important for prostate cancer progression. Overall, our results argue that chronic hypoxia and castration resistance commonly show alteration in the expression of metabolism-related genes part of the one-carbon and glycolysis-related pathways.

**Fig. 3:**
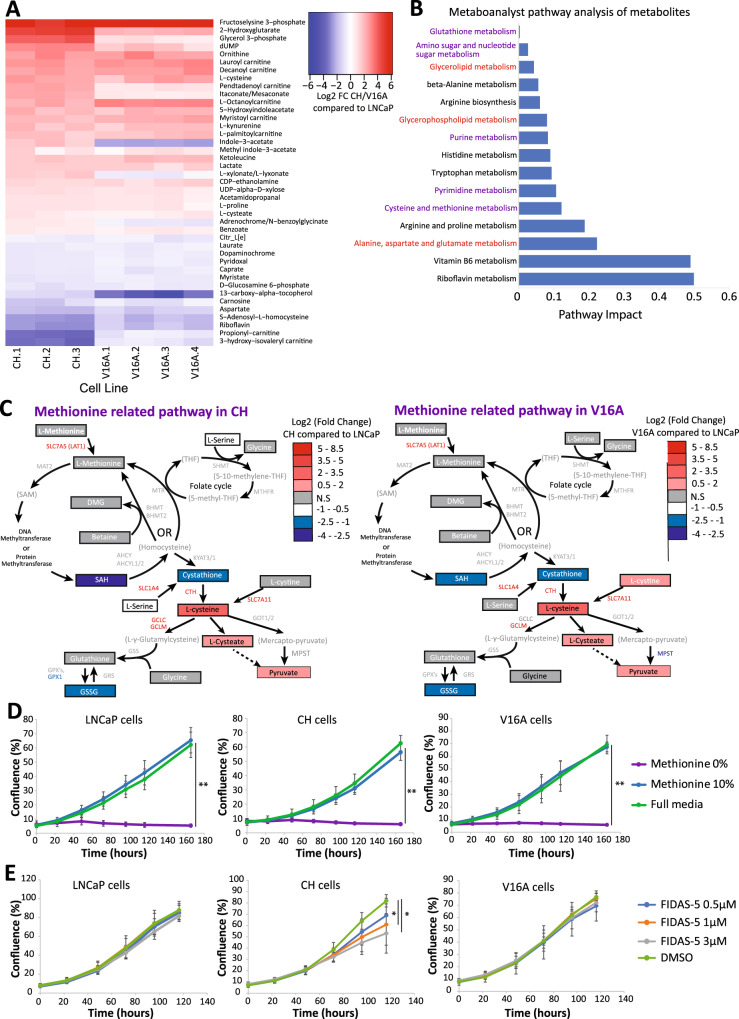
Metabolite changes implicate the methionine and related pathways. **A** A heat map of the shared significantly different metabolites in the LNCaP-CH and LNCaP-V16As when compared to LNCaP cells. There were 40 shared metabolites with a *q* = <0.05 and a minimum log2 fold change cut-off of ≥1.5. **B** Metaboanalyst pathway impact analysis of the 37 metabolites changed in the same direction. Purple text highlights pathways related to methionine and one carbon while the red text highlights glycolysis related pathways **C** Schematic pathway representation of shared methionine and one carbon related pathways in the LNCaP-CH and LNCaP-V16A cells. Metabolites, if included in metabolic profiling are in boxes and if not are in brackets. The log2 fold change is shown in each schematic with red indicating significantly increased, blue indicating significantly decreased and grey indicating no significant change in reference to the LNCaPs. The transcripts related to the pathways are coloured red for significantly increased, blue for significantly decreased and grey if not significantly different to the LNCaP cells. Moreover, the transcripts relating to each step of the pathway are located next to arrows to indicate involvement in each step of the pathway. The LNCaP, LNCaP-CH and LNCaP-V16A cells were cultured in media that was depleted of metabolites necessary for the methionine pathway, including L-methionine, L-cystine, Serine and Glycine. *T*-tests were performed to indicate significant differences in proliferation (*p value 0.05–10^−3^, ** 10^−3^–10^−6^) with the removal of the metabolites. **D** Represents proliferation of the LNCaP, LNCaP-CH and LNCaP-V16A cells in conditioned media that either contains 100% of metabolites required for the methionine pathway, (Full media), 10% of the metabolites required (Methionine 10%) or none of the metabolites required for the methionine pathway (Methionine 0%). All other metabolites required for survival of the cells were included in the media. **E** Represents growth of the LNCaP, LNCaP-CH and LNCaP-V16A cells in media containing either DMSO (control compound) or the MAT2A inhibitor FIDAS-5 at 0.5 μM, 1 μM or 3 μM.

We next directly assessed the impact of chronic hypoxia and castration resistance on metabolism by quantifying 301 metabolites in LNCaP-CH, LNCaP-V16A and LNCaP cells through LC-MS. In comparison to LNCaP cells, 95 intracellular metabolites were significantly different in LNCaP-CH cells (fold change ≥ 1.5, FDR of ≤0.05) (Supplementary Fig. 3A). In agreement with the gene expression analysis, these metabolites are enriched in glycolysis-related pathways, including pentose phosphate, pyruvate metabolism and the Citrate cycle (Supplementary Fig. 3B). This analysis also revealed increased levels of metabolites related to the methionine and glutathione pathways (Supplementary Fig. 3B). Similarly, 88 intracellular metabolites were significantly different in LNCaP-V16A compared to LNCaP cells (fold change ≥1.5, FDR ≤ 0.05) (Supplementary Fig. 3C). Across LNCaP-CH and LNCaP-V16A cells, 40 significantly altered intracellular metabolites were consistently altered except for Indole-3-acetate, L-xylonate and Adrenochrome, in both models (Fig. 3A). Focusing on the 37 shared metabolites identified metabolic disruptions to glutamate and fatty acid metabolism (Fig. 3B). It also revealed enrichment within the methionine, glutathione, purine, and pyrimidine pathways (Fig. 3B). The comprehensive characterisation of changes in the levels of metabolites and gene expression relevant to the methionine pathway revealed commonalities between LNCaP-CH and LNCaP-V16A cells versus LNCaP cells that extend to the folate cycle and the glutathione pathway (Fig. 3C). These data suggest methionine usage in the LNCaP-CH and LNCaP-V16A cells has been altered. Assessing the extracellular metabolites for changes in the availability of methionine pathway metabolites showed no difference in L-Methionine and L-Cystine, variable changes in L-Serine with only Glycine significantly decreased in the LNCaP-V16A cell culture media when comparing the media of the LNCaP-CH, and LNCaP cells (Supplementary Fig. 4A). To determine whether LNCaP-CH and LNCaP-V16A cells share an increased dependence on these metabolites we assessed their growth in methionine pathway depleted metabolite culture media. Unexpectedly, no differences in growth responses were observed across LNCaP-CH, LNCaP-V16A and LNCaP cells in low-methionine (10%) or no-methionine (0%) conditions. All cells were intolerant to methionine depletion and tolerant to low-methionine conditions (Fig. 3D). As cells can recycle methionine through the methionine and folate cycle an inhibitor of MAT2A was tested [44]. MAT2A is one of the enzymes in a complex that is responsible for the conversion of L-Methionine into S-adenosylmethionine (SAM), the universal methyl donor employed for DNA and protein methylation [45]. Only LNCaP-CH cells demonstrated some sensitivity to the MAT2A inhibitor FIDAS-5, in contrast to LNCaP-V16A and LNCaP cells which were insensitive to this inhibitor (Fig. 3E). These results argue that the LNCaP cell line, when exposed to chronic hypoxia, may rely on SAM production more than castration sensitive and resistant prostate cancer cell line models.

### Chronic hypoxia and castration resistance underlies Increased dependency on glucose uptake

**Fig. 4:**
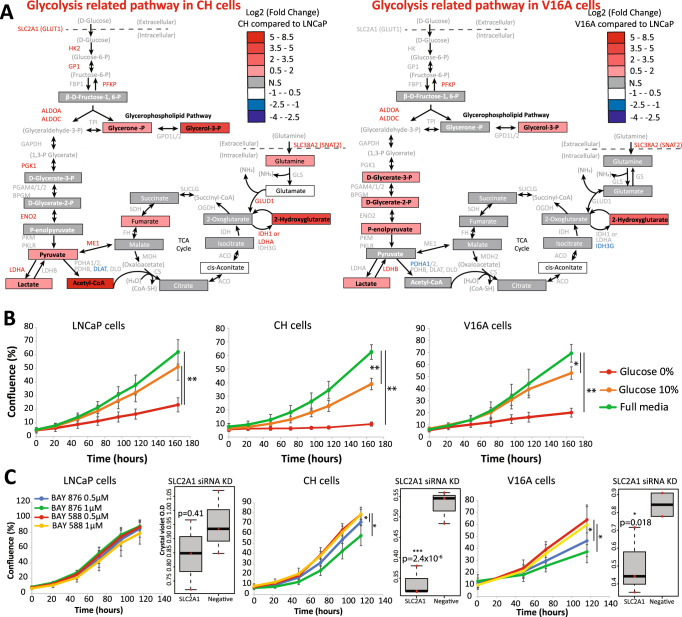
Metabolic changes in glycolysis identify a druggable weakness in the androgen insensitive cells. **A** Schematic representation of the glycolysis and related pathways in either the LNCaP-CH or LNCaP-V16A cells. The metabolites that were included in the metabolite analysis are in boxes while those not included are in brackets. Significantly increased metabolites are red, significantly decreased are in blue and those that were not significantly different to LNCaP cells are in grey (*q* < 0.05, log2 fold change ≥1.5). The genes involved in these pathways are either red for significantly increased or blue if significantly decreased in comparison to LNCaP cells and grey if there is no significant difference. **B** LNCaP, LNCaP-CH and LNCaP-V16A cells were cultured in conditioned media with proliferation monitored over time. The media was depleted of metabolites necessary for glycolysis and related pathways including Glucose, Glutamine and Glutamate. 100% of metabolites required for the glycolysis and related pathways, (Full media), 10% of the metabolites required (Glucose 10%) or none of the metabolites required for the glycolysis and related pathways (Glucose 0%). Unpaired *t*-tests were performed in each media condition to determine significant differences in proliferation (*p value 0.05–10^−3^, ** 10^−3^–10^−6^). **C** From these results the cells were exposed to an inhibitor of the glucose transporter (GLUT1) BAY-876 and the control compound BAY-588. Significant differences in proliferation were identified using unpaired *t*-tests. The right panel shows the effect of siRNA knock down (KD) compared to the scramble negative control (Negative) on viability of the cells using crystal violet staining.

Further interrogation of the disrupted metabolic pathways identified glycolysis related pathways in both the LNCaP-CH and LNCaP-V16A cells compared to LNCaP cells. Increased expression of genes related to these pathways was also observed at the transcriptional level. This argues for dependencies on glycolysis related pathways for castration resistance (Fig. 4A). To assess these dependencies, we first measured the abundance of glycolysis related metabolites in the cell culture media. Our results show reduced glucose and glutamine levels and increased glutamate levels in the media of all cells compared to the naive culture media (Supplementary Fig. 4B). While lower levels of glucose were observed in the media of the LNCaP-CH and LNCaP-V16A cells compared to LNCaP cells, this difference only reached significance in LNCaP-V16A cells (Supplementary Fig. 4B). We next looked for dependencies on glucose uptake for growth across our prostate cancer models using glucose pathway depleted culture media. Our results showed how LNCaP-CH and LNCaP-V16A cells are more sensitive to low-glucose (10%) as well as no-glucose (0%) conditions than the LNCaP cells (Fig. 4B). We further tested the sensitivity of our prostate cancer models to the GLUT1 inhibitor, BAY-876. While LNCaP cells were intrinsically resistant to GLUT1 inhibition, LNCaP-CH and LNCaP-V16A cells showed a dose-dependent significant growth inhibition upon treatment with the GLUT1 inhibitor BAY-876, in contrast to BAY-588, the negative control compound which is 600 times less active (Fig. 4C) [46]. Furthermore, knockdown of the SLC2A1 gene (GLUT1) using siRNA was able to confirm the dependency of the LNCAP-CH and LNCaP-V16A cells on the glucose transporter (Fig. 4C, Supplementary Fig. 4C). Collectively, our results argue for a shared dependency on glucose metabolism in prostate cancer cells chronically exposed to hypoxia and independently established models of castration resistance. Furthermore, these results support the inclusion of GLUT1 chemical inhibitors in the development of treatment strategies to limit prostate cancer progression.

## Discussion

Here, we demonstrated how chronic hypoxia favours androgen insensitivity in prostate cancer cells, a feature central to the progression to lethal castration resistant prostate cancer. Our results expand on previous studies reporting the role for hypoxia to increase migration and invasion properties in prostate cancers [47, 48], features typical of the response to hypoxia across various cancer types [25]. Furthermore, these results parallel the impact reported from cycles of acute hypoxia toward androgen insensitivity in prostate cancer [26, 49]. Although acute hypoxia has been implicated in prostate cancer progression [50], in vivo evidence suggests chronic hypoxia increases prostate tumour growth [4]. Moreover, longer exposure to hypoxia (>24 h) progressively decreased the expression of RAD51, required for homologous recombination (HR) during double strand break repair, in prostate cancer [51]. A loss of HR in the cells leads to increased genomic instability [51]. Therefore, the longer cells are exposed to hypoxia the more likely they are to become genetically unstable, promoting tumour genetic heterogeneity, observed in hypoxic prostate cancer samples [20] and an attribute of metastatic CRPC [52]. Collectively, our work proposes a contribution of chronic hypoxia towards increased risk of progression by favouring androgen insensitivity in prostate cancer.

We further demonstrate how chronic hypoxia alters transcriptional and metabolic pathways; mimicking changes observed in castration-resistant prostate cancer cells. We specifically identify transcriptional and metabolic changes affecting the methionine pathway. The methionine pathway accounts for the production of S-adenosylmethionine (SAM), the universal methyl donor for protein and DNA methylation [45]. Our results show a decrease in the abundance of S-adenosylhomocysteine (SAH), a potent inhibitor of methylation formed from the donation of the SAM methyl group to a methyltransferase, implying a change in the usage of SAM [45, 53]. However, we could not detect increased sensitivity to short term methionine depletion in prostate cancer cells following chronic exposure to hypoxia. This may be, in part, due to histone methylation acting as a sink for the methyl group, indicating short-term depletion may have little effect on the cells [54]. However, the subsequent investigation of the MAT2A inhibitor, the enzyme responsible for the conversion of L-methionine to SAM [45], only identified a susceptibility in prostate cancer cells maintained under chronic hypoxia. These findings suggest the hypoxic cells have a greater dependence on the production of SAM from L-methionine, which may reflect a greater dependence of glutathione production for reactive oxygen detoxification [55]. Collectively, these results suggest that chronic hypoxia induces changes in the methionine cycle typical of those observed in castration-resistant prostate cancer. Furthermore, altering this pathway may lead to changes in methionine reservoirs within the hypoxic and castration-resistant cells that could be further investigated and therapeutically exploited.

Beyond the methionine pathway, we observed significant differences in glycolysis-related pathways that suggest an increased dependency on glucose under chronic hypoxia exposure and castration-resistant prostate cancer cells. Work by Geng et al. demonstrated cycles of acute hypoxia promoted the transcription of *GPI*, which promoted a metabolic shift to glycolysis [26]. The changes in these metabolic pathways reduced the sensitivity of these cells to enzalutamide, an AR inhibitor. The silencing of *GPI* resensitized the cells to enzalutamide [26]. The transcriptional analysis with LNCaP-CH cells also identified *GPI* expression as increased during chronic hypoxia. However, *GPI* was not differentially expressed in the LNCaP-V16A cells (Fig. 4A). Indicating therapeutically targeting or using increased expression of *GPI* alone does not identify androgen insensitive cells. However, the shared transcriptional changes in the hypoxic and castration-resistant cells identified enzymes involved in the early stages of the glycolysis pathway, with the hypoxic cells showing changes in glucose membrane transporter GLUT1, a marker of hypoxia [56], and a known prognostic factor for recurrence in prostate cancer [57]. Moreover, the preferential expression of the hypoxic and castration resistance gene set within the Cases group of the Porto cohort and the identification of ZNF560 in the CPC-Gene cohort demonstrate there is a chronic hypoxia mechanism involved in prostate cancer progression. The increased dependence on glucose metabolism during prostate cancer progression has implications for treatment [43]. However, the data presented here implies both the transcriptional and metabolic analysis were required to fully identify the dependency on glycolysis. The metabolic analysis further implicated a dependency on glucose uptake in androgen insensitivity. In agreement, we report a vulnerability to the GLUT1 inhibitor BAY-876 in both chronic hypoxia and castration-resistant prostate cancer cells, revealing a therapeutically targetable weakness arising under chronic hypoxia.

## Methods

### Cell lines—media, 1% oxygen chamber, Incucyte, compound/drug testing

#### Cell lines

LNCaP clone FGC (ATCCⓇ, CRL-1740^TM^), VCaP (ATCCⓇ, CRL-2876™) RWPE-1 (ATCCⓇ, CRL-11609^TM^), PWR-1E (ATCCⓇ, CRL-11611^TM^) and the V16A cell line (gifted from Hansen He, originally from Amina Zoubeidi) were used in the subsequent experiments.

#### Cell culture maintenance conditions

LNCaP cells were cultured in RPMI (ThermoFisher, #11875093) with 10% fetal bovine serum (FBS) (ThermoFisher Scientific, #12483020) and Penicillin/Streptomycin (10,000 U/ml, ThermoFisher Scientific, #15140122) at 21% oxygen or Penicillin/Streptomycin/Amphotericin B (100x, ThermoFisher Scientific, #15240062) at 1% oxygen. VCaP cells were maintained in DMEM (ThermoFisher Scientific 11995065) with 10% FBS (ThermoFisher) and Penicillin/Streptomycin (ThermoFisher Scientific) at 21% oxygen or Penicillin/Streptomycin/Amphotericin B (ThermoFisher Scientific) at 1% oxygen. The V16A cell line (derived from LNCaPs, and subsequently referred to as LNCaP-V16A) was cultured in RPMI-phenol red media (ThermoFisher Scientific, #11835030) with 10% charcoal depleted FBS (Wisent Cat: 080750, Lot: 912070), Penicillin/Streptomycin and were maintained at 21% oxygen. The RWPE-1 and PWR-1E cell lines were cultured in keratinocyte serum-free media (KSF, Thermofisher Scientific, #17005042) supplemented with recombinant epidermal growth factor (EGF) and bovine pituitary extract (BPE). Cells were counted using the Countess™ cell counter (Invitrogen, #C10281).

#### Generation of the CH cells

LNCaP/VCaP cells were plated at a density of 30–50% and maintained in 1% oxygen (Whitley H35 hypoxystation) in biological triplicate. During IncucyteⓇ S3 (Sartorius) experiments, cells were briefly removed from hypoxia for imaging. Cells were maintained in 1% oxygen for a minimum of 144 h to be considered chronically hypoxic (CH) and were continuously cultured at 1% oxygen for all experimentation unless stated otherwise.

#### Cell culture experimental conditions

Cells cultured in RPMI media (ThermoFisher) with 10% FBS (ThermoFisher Scientific) and Penicillin/Streptomycin (10,000 U/ml, ThermoFisher Scientific) hereby referred to as Androgen + media. Cells were cultured in RPMI-phenol red media (ThermoFisher Scientific) with 10% Charcoal depleted FBS (Wisent) and Penicillin/Streptomycin (ThermoFisher Scientific), hereby referred to as Androgen - media. Enzalutamide (final concentration 10 µM, Axom, #1613) was added to the Androgen - media, making Androgen - with ENZA.

#### siRNA transfection conditions

For siRNA transfection, TriFECTa® RNAi Kits for AR (hs.Ri.AR.13), SLC2A1 (hs.Ri.SLC2A1.13), ZNF560 (hs.Ri.ZNF560.13) and the negative control (Negative Control DsiRNA), designed by Integrated DNA Technologies Canada, Inc. (IDT, Iowa, USA) siRNA were used at 30 nM concentrations. The siRNA was combined with Opti-MEM (Invitrogen, #31985062) and Lipfectamine RNAiMAX (Invitrogen, #13778150) as per manufacturer’s instructions in either a 24 (Corning, #3524) or 6 well plate (Corning, #3516). Crystal violet staining was measured from technical duplicates generated from biological replicates [2, 3] in cells harvested 72 h after siRNA transfection. Optical density was then measured at 562 nm. RNA and protein were collected at the 72 h post transfection time point.

#### Conditional medium experimental conditions

For medium testing LNCaP, LNCaP-CH and LNCaP-V16A cells were plated in 96-well plates (Millipore Sigma, CorningⓇ CLS3596–50EA) in biological triplicate. RPMI media depleted of metabolites for the methionine pathway and glycolysis was synthesised by Wisent (350–055-CL, custom order). A base media was made with the RPMI metabolite depleted media, charcoal depleted FBS and Penicillin/Streptomycin to be consistent with the RPMI - steroid base media. Each of the metabolites required was supplied at 100x concentration, except for D-Glucose, which was supplied at 75x concentration (Wisent, custom order).

#### Chemical compound treatment

LNCaP, LNCaP-CH and LNCaP-V16A cells were plated in 96-well plates (Millipore Sigma, CorningⓇ CLS3596–50EA) in biological replicate [2, 3] and technical duplicate. The chemical compounds BAY-876 (SGC, Toronto), BAY-588 (SGC, Toronto), V-9302 (Aobious, #AOB33597) and MAT2A Inhibitor II (FIDAS-5, Calbiochem, Millipore Sigma 5041730001) were diluted in DMSO (Bioshop DMS666.100) to 10 mM stock solutions. The chemical compounds were added at 2x concentration in 100 µl of media to each well. An equal volume of the control DMSO (Bioshop DMS666.100) was added to the 100 µl of media for MAT2A control. Cells were continuously monitored at 21% oxygen using the IncucyteⓇ S3 (Sartorius).

### Western blotting

For hypoxic cell western blots cells were incubated in 1% oxygen for varying lengths of time, from 8 to 144 h, and collected in ice-cold DPBS (ThermoFisher Scientific, #14190144) immediately prior to subcellular fractionation, performed using the NE-PER Nuclear and Cytoplasmic Extract Kit (ThermoFisher Scientific, #0078833). For assessment of knock-down, cells were incubated for 72 h with specific siRNAs before collection into RIPA (25 mM Tris-HCl pH 7.6, 150 mM NaCl, 1% NP-40, 1% sodium deoxycholate, 0.1% SDS) protein lysis buffer, then incubated on ice before centrifugation at 20,000 g for 10 min at 4 °C. Protein was quantified using the Pierce™ BCA protein assay kit (ThermoFisher Scientific, #23227) and 20 µg of the nuclear fraction (NF) from the NE-PER or 25 µg of whole cell lysate (WCL) collected using RIPA was prepared by adding loading dye (5x concentration, 250 mM Tris-HCl ph 6.8, 10% SDS, 50% Glycerol, 500 mM DTT, 0.05% Bromophenol Blue) to a final concentration of 1x. Protein samples were boiled for 10 min at 95 °C then run on an Any kD^TM^ Min-iPROTEANⓇ TGX^TM^ precast gel (Bio-rad, 10 well, #4569034) and transferred using a Trans-Blot Turbo Midi 0.2 µm PVDF (Transfer packs, Bio-Rad #1704157) system to PVDF membrane. The antibodies used were HIF1ɑ (1:500, Abcam, #ab2185), AR (1:1000, Epicypher, #13–2020), ZNF560 (1:500, Invitrogen, #PA5–41224), H3 (1:10,000, Abcam, #ab1791) and nuclear protein control TBP (1:1000, Cell Signalling, #44059 S) with an anti-Rabbit (1:2500, Li-Core, #926–32213) secondary antibody. Membranes were imaged using ODYSSEY™ CLx (Li-Cor) and images analysed using Image Studio™ (Li-Cor).

### RT-qPCR

Cells were plated in 6-well plates (Cellstar, #657160) and either incubated at 21% oxygen or 1% oxygen. RNA was collected and purified using the Allprep DNA/RNA mini kit (Qiagen, #80204), as per manufacturer’s instructions. RNA was quantified via NanoDrop (NanoDrop 2000, ThermoFisher Scientific, ND2000CLAPTOP). After quantification 1 µg of RNA was used for cDNA synthesis, performed using the SensiFAST™ kit (Meridian Bioscience, #BIO-65054). HIF1ɑ specific gene set selected from Semeza GL., [23] and Beyer et al. [31]. The AR target gene was selected from Wilson et al. [30]. DNA oligonucleotide primers were synthesised by Integrated DNA Technologies Canada, Inc. (IDT, Iowa, USA) (Supplementary Table 1). Quantitative PCR (qPCR) was performed using SensiFAST™ SYBR® No-ROX Kit (Meridiant Bioscience, #BIO-98050) on a BIORAD c1000 touch thermocycler with the CFX96 touch^TM^ Real-time detection system (BioRad). All qPCR results were analysed in Excel (Microsoft) as the log 2-fold change of the ΔΔCt (2^-(ΔΔCt)) using the expression of TBP as the control transcript. To measure changes in HIF1ɑ target transcripts all time points, measured at either 21% oxygen or 1% oxygen, were normalised to the 0 time point of each transcript.

### RNA sequencing and data processing

#### Sample preparation

Biological replicates of each cell line were plated in 6-well plates and cultured in RPMI - steroid + DHT or RPMI - steroids, depending on the cell type, for 72 h. The cells were washed two times in DPBS and then placed on ice. The RNeasy plus mini kit (Qiagen, #74136) was used to collect RNA from cells as per manufacturer’s instructions. RNA was then quantified using the Nanodrop and 1 µg of RNA was delivered to the Princess Margaret Genomics Centre (PMGC, Toronto, Canada). The PMGC performed ribosomal RNA depletion using Ribo-Zero Gold rRNA Removal kit (Illumina).

#### Sequencing

The RNA libraries were sent for 75 bp paired-end sequencing on an Illumina NextSeq 550 at a target depth of 60 M read pairs per sample. Sequence quality was assessed with FastQC (v0.11.8) [58], and FASTQ files from multiple lanes corresponding to the same samples were merged. Reads were trimmed for quality with TrimGalore! (v0.6.2) [59] using Cutadapt (v2.3) [60]. Trimmed read pairs were used to quantify transcripts with Kallisto (v0.45.1) [61] against the GRCh38 transcriptome reference index (Ensembl v94) with the following command: kallisto quant -b 100 -t 8 --pseudobam -i {index} {sample_R1} {sample_R2}. One of the three V16A replicates was removed at this point, due to more than 75% of transcripts having 0 compatible reads assigned to them.

#### Differential gene expression analysis

To remove sequencing batch effects between samples, the SVA model (Bioconductor R package v3.36.0 [62]) was used to calculate one surrogate variable in R (v3.5.1) [63]. Transcript counts were modelled with Sleuth (v0.30.0) [64], using a contrast model consisting of the cell type, sequencing batch, and surrogate variable calculated from SVA. Only transcripts where > = 60% of samples contained > = 10 reads were considered. The Wald test was performed to calculate significantly differentially expressed transcripts, and results were aggregated on a gene-level basis. Multiple *p* values were adjusted using the Benjamini–Hochberg FDR method. Differentially expressed transcripts or genes were considered significant if the FDR values were <0.05.

#### Gene score analysis

Gene Ontology (GO) term and Kyoto Encyclopedia of Genes and Genomes (KEGG) pathway analysis were performed using the clusterProfiler R package [65] using the “fdr” pAdjustMethod with a minimum gene size of 1 as the only modification to default settings.

### Metabolite extraction

#### Sample preparation

Biological replicates of each cell line were plated in 6 well plates and cultured in respective media for 72 h. The LNCaP and LNCaP-CH were cultured in RPMI - Steroid + DHT and the V16A were cultured in RPMI - Steroid. The cells were collected at approximately 70% confluence and counted to ensure there were between 5 × 10^5^ and 1 × 10^6^ cells (Supplementary Table 2). For conditioned media collection, 1 mL of media was removed from each biological replicate. Naive media, in technical replicates, were collected in parallel with conditioned media. Cells were washed 2 times in 0.9% sodium chloride in HPLC grade H_2_O (Caledon, #8801–7–40) solution and placed on ice. 1 mL of 80% HPLC grade Methanol (Caledon, #6701-7-40), previously stored at −80 °C, was added to each well with plates then placed at −80 °C for 15 min. Once removed from −80 °C the plates were placed on dry ice and cells were scraped into 1.5 mL tubes. The cell suspension was then centrifuged at 20,000 g for 10 min at 4 °C. The supernatant was then divided into 2 separate 1.5 mL tubes with approximately 450 µl of extract in each and either stored at −80 °C or dried with Vacufuge® plus (Eppendorf). Samples dried via Vacufuge® plus were stored at −80 °C prior to shipping. Liquid chromatography-mass spectrometry (LC-MS) of metabolite extracts and media samples was performed by Juan Lui with supervision from Jason Locasale (Duke University, NC, USA) as previous published [66].

#### Analysis

The peak intensity of the LC-MS was used for all analysis and reading were excluded if the value read 1000 as this indicated lack of detection. The intracellular metabolites were normalised first by quantile normalisation, then log2 transformed and mean centred. For the media (extracellular) metabolites the samples were quantile normalised. The LNCaP cell line samples were used as the control condition for all subsequent analyses. The intracellular metabolites for the LNCaP-CH and LNCaP-V16As were compared to the LNCaPs using an unpaired *t*-test. The cut-off for significantly different was set at 1.5-fold change with a false discovery rate (FDR) of 0.05. For the media (extracellular) analysis the naive media replicates were combined to set the naive media peak intensity level. The conditioned media for all samples were compared to the naive media for unpaired *t*-test analysis.

### CH and V16A shared transcript profile in patient samples

The expression of the 199 genes commonly up-regulate in LNCaP-CH and LNCaP-V16A compared to LNCaP cell lines was assessed across primary prostate tumours from the Porto Cohort to calculate a gene score [43]. The gene score reported for each patient corresponds to the sum of the 199 genes expressed above (+1) or below (−1) their mean expression across all tumours from the Porto Cohort. The gene scores were then compared between patients that develop biochemical recurrence (Cases) versus those that don’t (Controls). This was achieved by taking the mean of the expression score of each gene of the 166 identified out of the 199 gene set. The data was then separated into the top and bottom 50th percentile before use in a box plot and statistical analysis in R (v1.4.1717). A *t*-test with FDR correction was performed on the case and control group scores to determine any statistical differences. For the investigation of transcription factors in prostate cancer patient data the CPC-Gene cohort [1] was used for the Kaplan-Myer and survival hazard ratio [67] analysis.

## Supplementary information

Supplementary figure and table legends

Supplementary Figure 1

Supplementary Figure 2

Supplementary Figure 3

Supplementary Figure 4

Supplementary Table 1

Supplementary Table 2

## Data Availability

RNA-seq data generated in this study were deposited in the NCBI GEO database under accession number GSE227811.
