# Supplementary material for: Chronic hypoxia favours adoption to a castration-resistant cell state in prostate cancer
Source: Oncogene. 2023 Apr 5;42(21):1693–703. doi: 10.1038/s41388-023-02680-z (PMC10202808; doi:10.1038/s41388-023-02680-z)
Supplement: Supplementary file 1 — Supplementary figure and table legends [file 41388_2023_2680_MOESM1_ESM.docx]

**Supplementary Figure Legends**

Supplementary Figure 1

A) VCaP cells were cultured in either 21% (blue) or 1% oxygen (red), with N.S representing no significant difference. B) Proliferation at 21% and 1% oxygen of two immortalised prostate derived cell lines, the RWPE-1 and PWR-1E (* p value=0.05 – 10^-3^, ** p value=10^-3^ - 10^-6^, *** p value=>10^-6^) was monitored over time. C) The expression of HIF1ɑ target genes *SLC2A1* and *KDM3A*, normalised to TBP, in the VCaP cells. D) Proliferation of the VCaP-CH cells when reoxygenated (21%) was compared to cells continuously grown in 1% oxygen. E) Shows the difference in proliferation when the VCaP and VCaP-CH cells were exposed to full RPMI media (Androgen +), RPMI – steroids (Androgen -) and RPMI – steroids + Enzalutamide (Androgen - with ENZA). F) Proliferation of the LNCaP-V16A cells, in Androgen - media, at either 21% or 1% oxygen. G) Western blot for the Androgen receptor (AR) in the siRNA knock down (KD) for AR and a scrambled negative control (Negative) with H3 as the loading control in whole cell lysate. H) qPCR analysis of the down-stream target of AR, *KLK3* (Prostate Specific Antigen).

Supplemental Figure 2

A) An upset plot representing the differentially expressed transcripts identified in the LNCaP-CH and LNCaP-V16A cells when compared to the LNCaP cells. B) A KEGG pathway analysis of the 199 shared up-regulated transcripts in the LNCaP-CH and LNCaP-V16A cells. C) A Venn diagram of the shared down-regulated transcripts in the LNCaP-CH and LNCaP-V16A cells when compared to the LNCaPs. The right panel shows the GO term analysis of the shared 310 down-regulated transcripts identifying only one pathway. D) GO term pathway analysis of the uniquely upregulated (Top panel) and uniquely down-regulated (Bottom panel) gene transcripts in the LNCaP-CH cells when compared to the LNCaPs. E) GO term analysis of the uniquely upregulated (Top panel) and uniquely down-regulated (Bottom panel) gene transcripts in the LNCaP-V16A cells when compared to the LNCaPs. F) The box plot represents the expression of 166 identified out of the 199 LNCaP-CH and LNCaP-V16A gene set identified in the Porto cohort. Samples were separated based on whether they were in the “Cases” (patients who progressed to BCR) or “Control” (patients who did not progress to BCR) group. Unpaired one-tailed t-tests with FDR correction were performed on the case and control groups to generate a q-value of 0.0729. G) The plot shows all transcription factors identified in the LNCaP-CH and LNCaP-V16A shared up-regulated gene transcripts, with the top 3 emphasised in larger text. H) Hazard ratio analysis of ZNF560 in the CPC-Gene patient cohort. I) Western blot for the ZNF560 siRNA knock down (KD) and scramble negative control (Negative) with histone 3 (H3) used as a loading control. The arrow indicates the ZNF560 protein band.

Supplementary Figure 3

A) A heat map of the significantly different (q value <0.05, log2 fold change ≥1.5) metabolites in the LNCaP-CHs compared to the LNCaP cells. B) Metaboanalyst pathway impact analysis of all significantly altered metabolites. Purple text highlights pathways related to methionine and one carbon while the red text highlights glycolysis related pathways. C) Heat map of the significantly altered metabolites (q value <0.05, fold change ≥1.5) in the LNCaP-V16A as compared to the LNCaP cells. D) A Metaboanalyst pathway impact analysis of the significantly altered metabolites in the LNCaP-V16A cells. With pathways colour coded as previously indicated.

Supplementary Figure 4

A) Bar graphs representing the quantile normalised peak intensity of the methionine related metabolites profiled in the media during the metabolic analysis (n = 3 for LNCaP-CH and 4 for LNCaP, LNCaP-V16A). The red dotted line indicates the naive media concentration of the metabolite with statistical significance determined by unpaired t-tests (* p value=0.05 – 10^-3^, ** p value=10^-3^ - 10^-6^, *** p value=>10^-6^). B) Bar graphs representing the quantile normalised peak intensity of the glycolysis related metabolites profiled in the media during the metabolic analysis (n = 3 for LNCaP-CH and 4 for LNCaP, LNCaP-V16A). C) qPCR for *SLC2A1* (GLUT1 protein) expression using siRNA knock down (KD) with scramble negative control (Negative).

Supplementary Table 1

Primers for qPCR expression analysis.

Supplementary Table 2

Cell counts for the LNCaP, LNCaP-CH (LNCaP LT1) and LNCaP-V16A cell lines used in the metabolic analysis.
