# Supplementary material for: Chronic hypoxia favours adoption to a castration-resistant cell state in prostate cancer
Source: Oncogene. 2023 Apr 5;42(21):1693–703. doi: 10.1038/s41388-023-02680-z (PMC10202808; doi:10.1038/s41388-023-02680-z)

Supplementary Figure 1

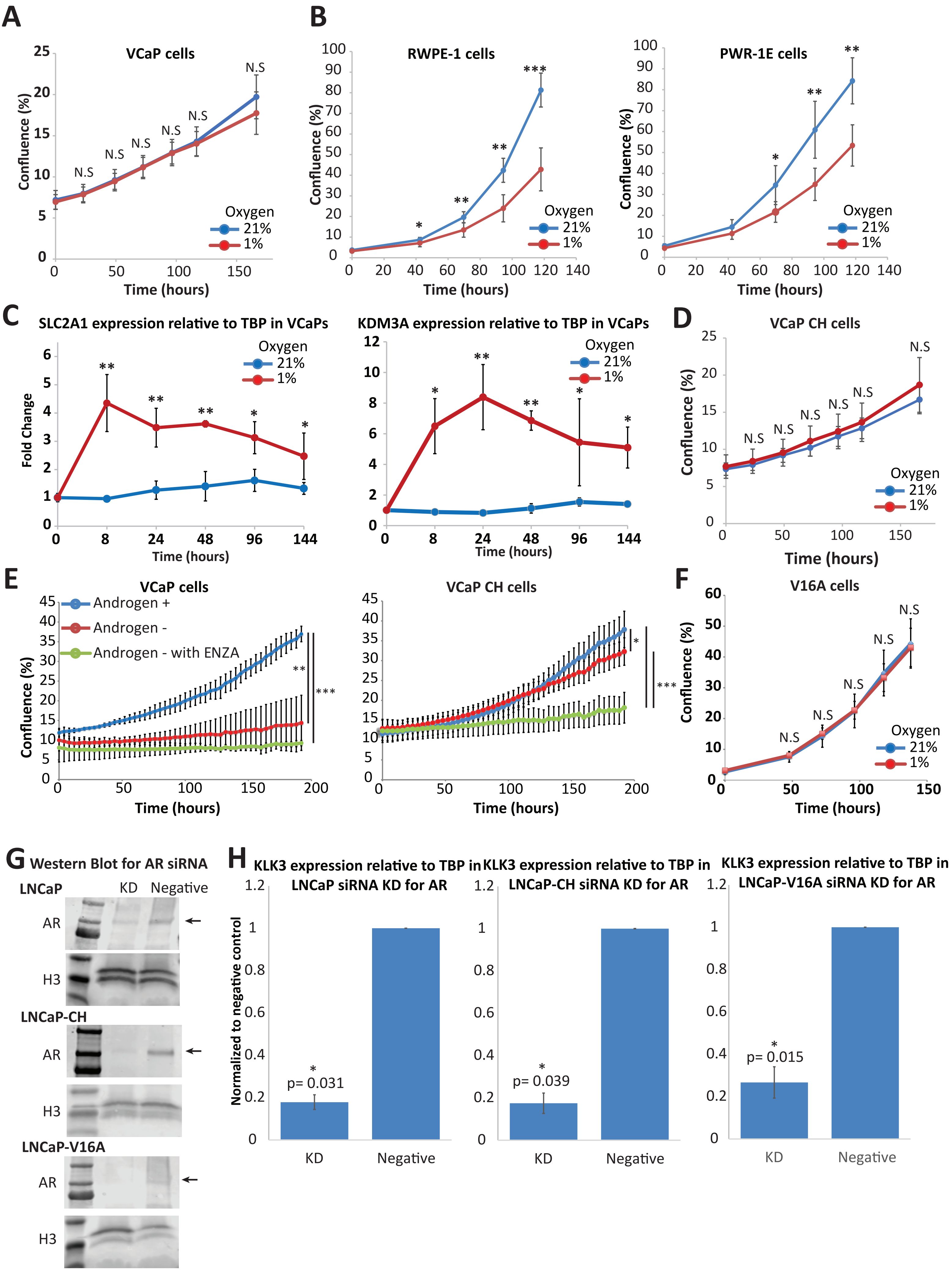

Supplement: Supplementary file 2 — Supplementary Figure 1 [file 41388_2023_2680_MOESM2_ESM.pdf]
