# Supplementary figures and images for: Chronic hypoxia favours adoption to a castration-resistant cell state in prostate cancer
Source: Oncogene. 2023 Apr 5;42(21):1693–703. doi: 10.1038/s41388-023-02680-z (PMC10202808; doi:10.1038/s41388-023-02680-z)

# A

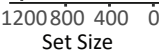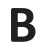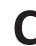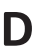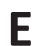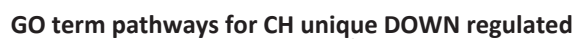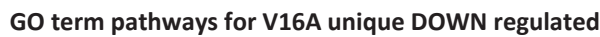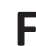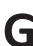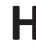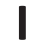

# Events: 17; Global p-value (Log-Rank): 0.029369

AIC: 139.29; Concordance Index: 0.71

Supplement: Supplementary file 3 — Supplementary Figure 2 [file 41388_2023_2680_MOESM3_ESM.pdf]

# Supplementary Figure 4

**A**

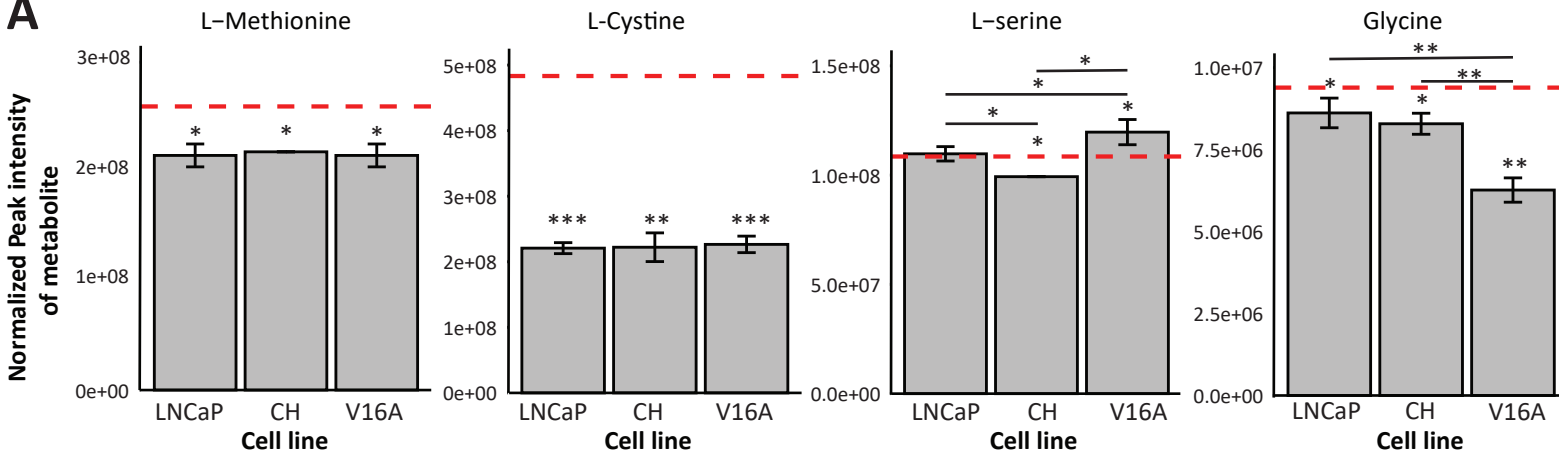

**B**

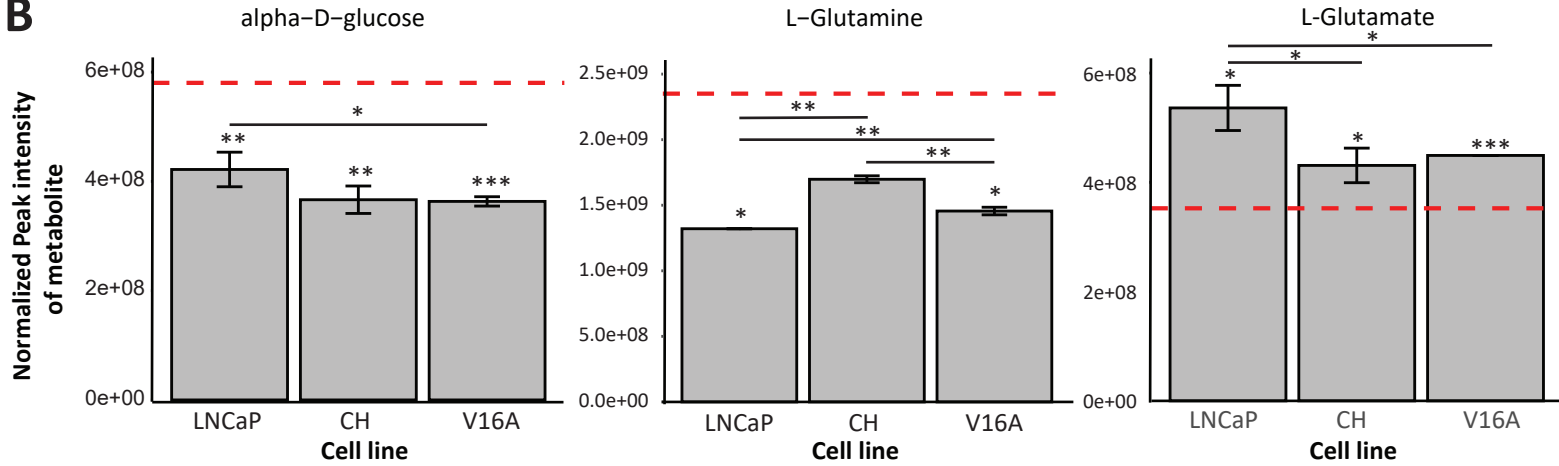

**C**

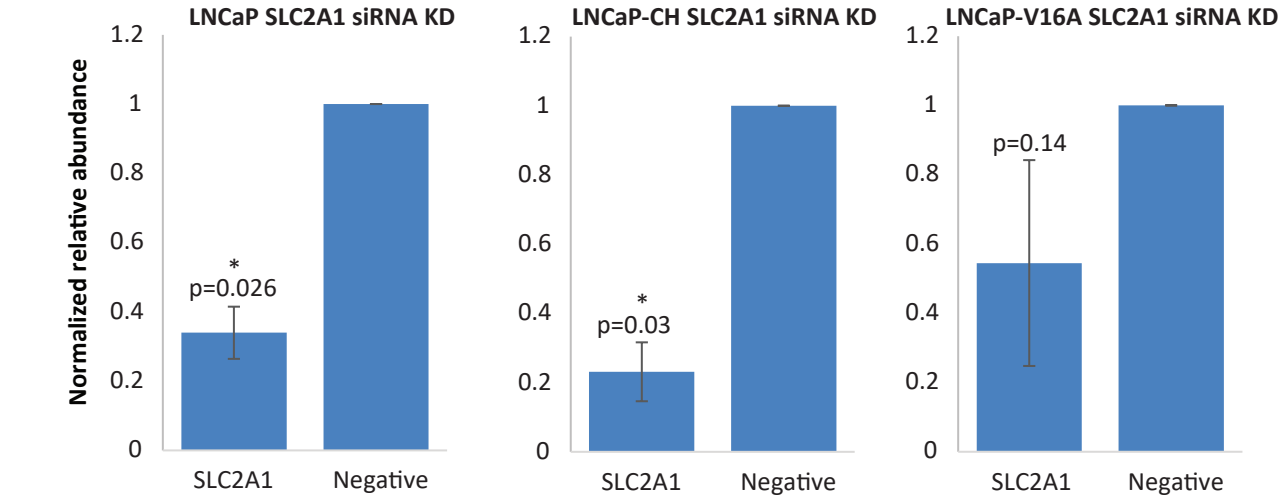

Supplement: Supplementary file 5 — Supplementary Figure 4 [file 41388_2023_2680_MOESM5_ESM.pdf]
