# Supplementary material for: Chronic hypoxia favours adoption to a castration-resistant cell state in prostate cancer
Source: Oncogene. 2023 Apr 5;42(21):1693–703. doi: 10.1038/s41388-023-02680-z (PMC10202808; doi:10.1038/s41388-023-02680-z)

# Supplementary Figure 3

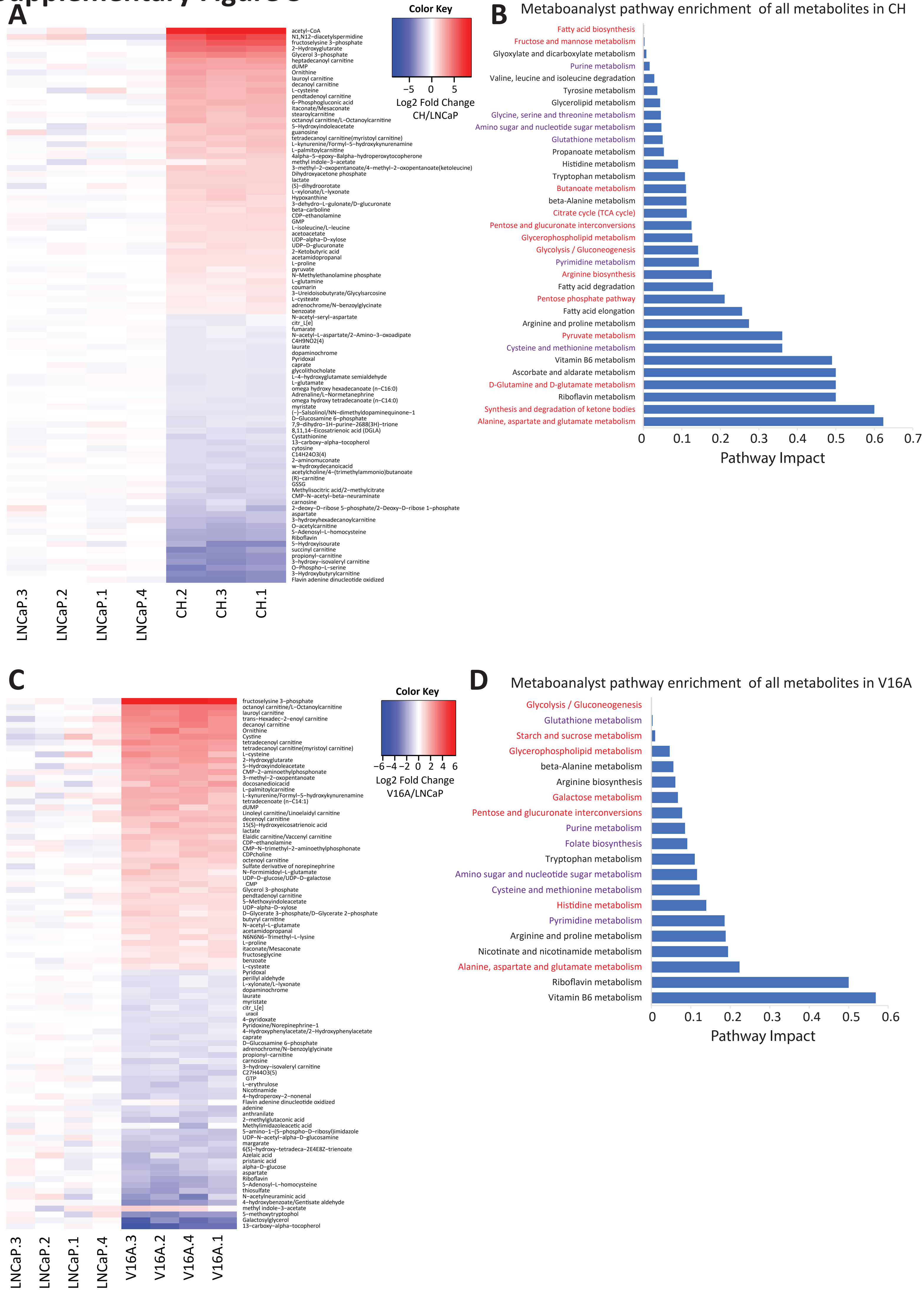

Supplement: Supplementary file 4 — Supplementary Figure 3 [file 41388_2023_2680_MOESM4_ESM.pdf]
